# Supplementary material for: Maize GOLDEN2-LIKE genes enhance biomass and grain yields in rice by improving photosynthesis and reducing photoinhibition
Source: Commun Biol. 2020 Apr 1;3:151. doi: 10.1038/s42003-020-0887-3 (PMC7113295; doi:10.1038/s42003-020-0887-3)

Fig. 2k – D1

0h-HL-H_2O

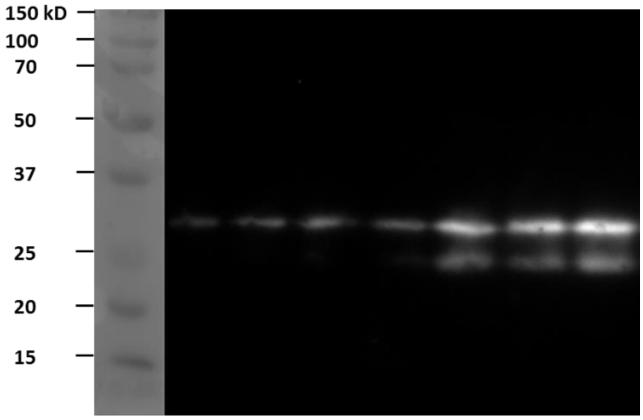

0h-HL-Lin

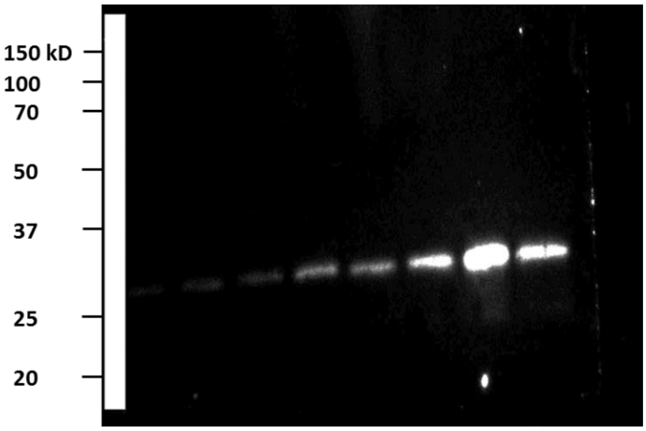

4h-HL-H_2O

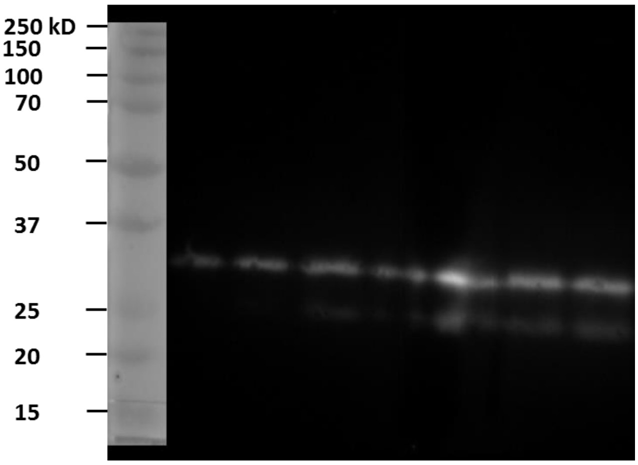

4h-HL-Lin

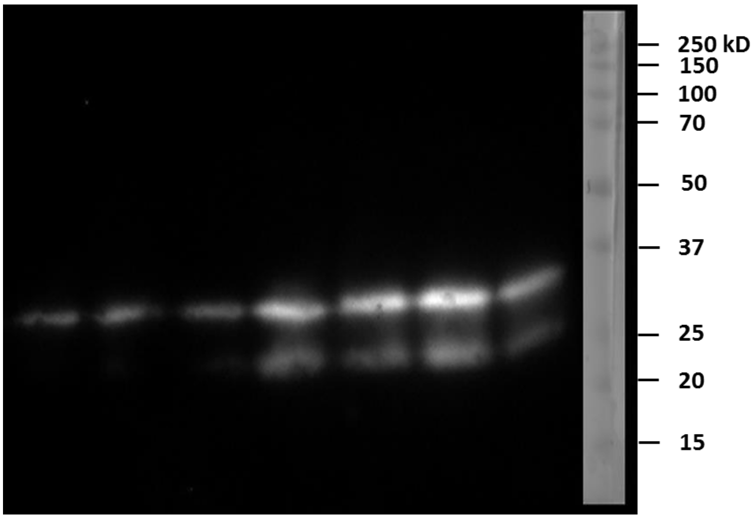

CBB

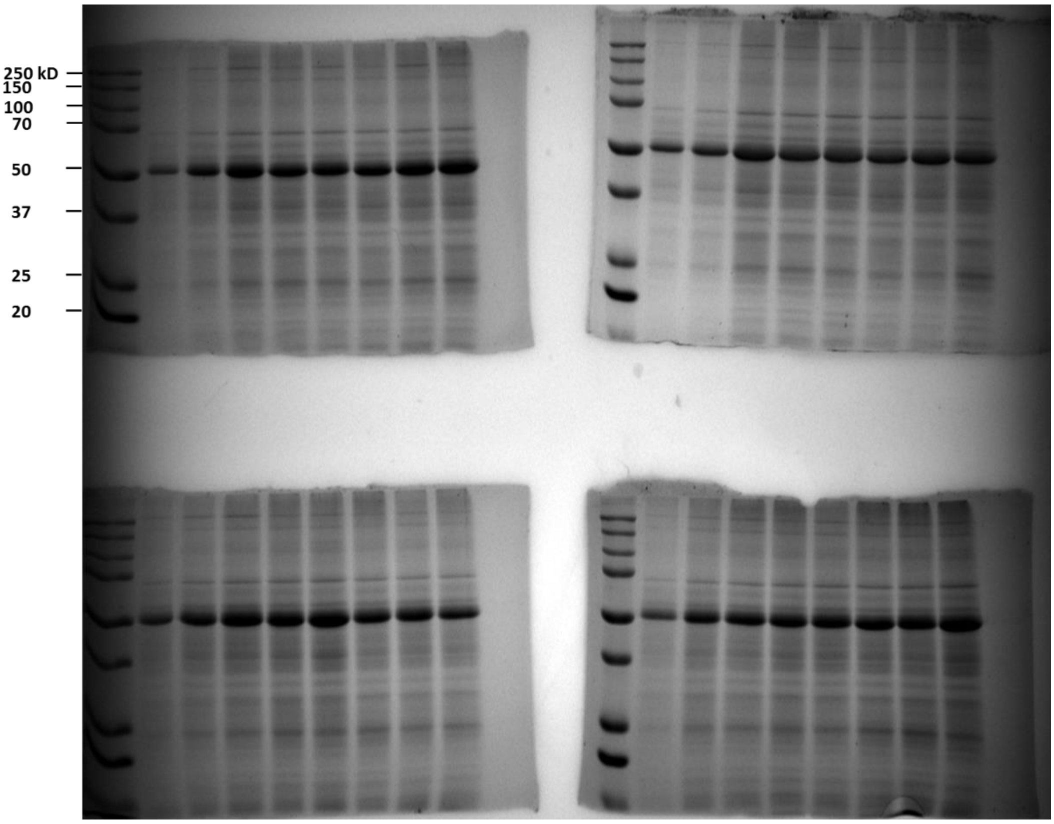

Supplementary Figure 2b

Lhca1

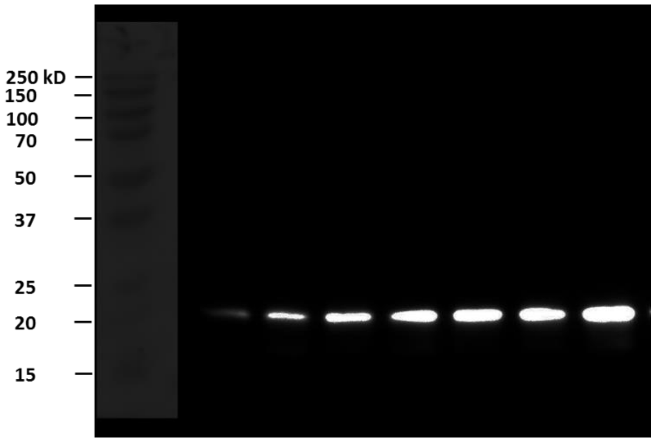

Lhcb2

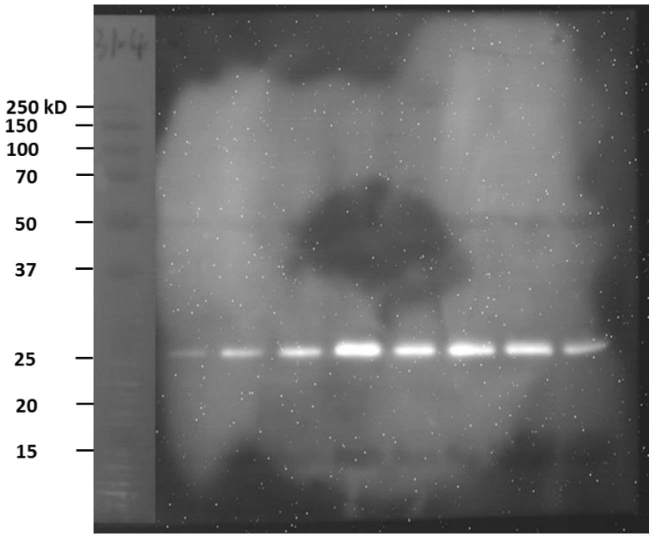

PsaA

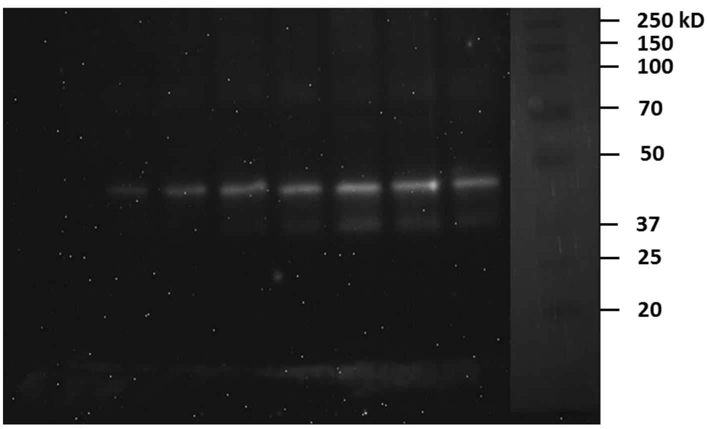

D1

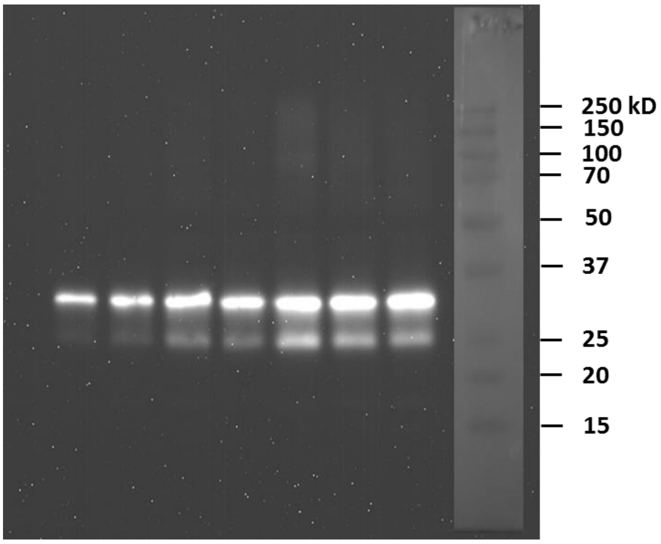

PsbS

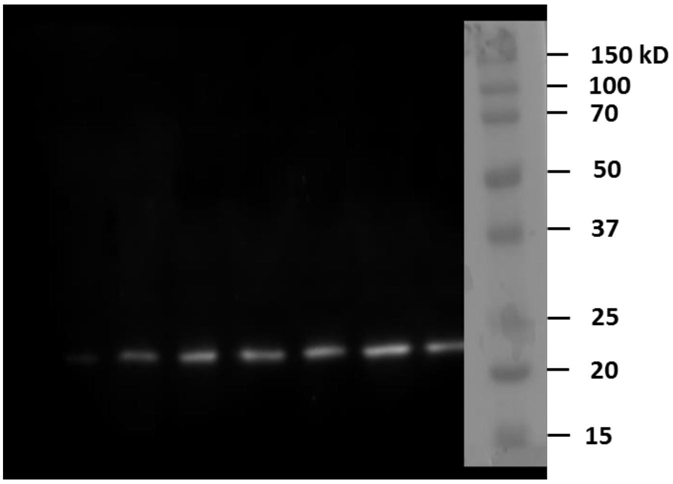

Cytb6

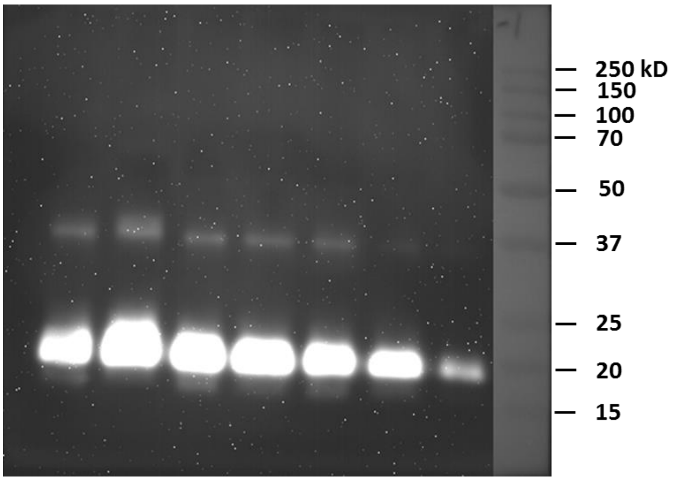

AtpA

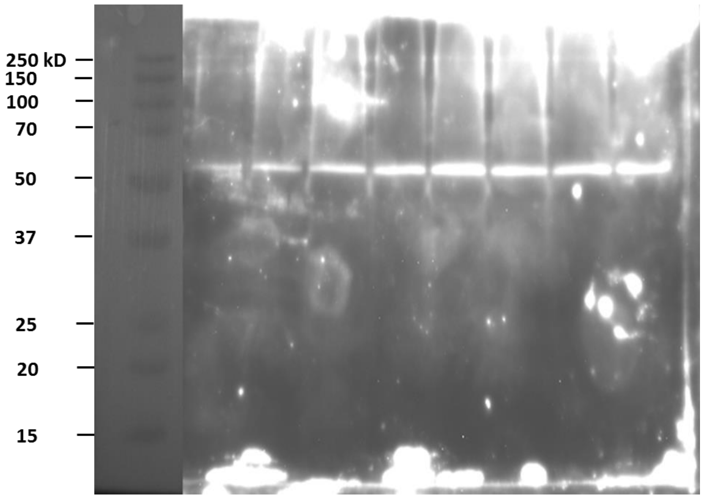

AtpB

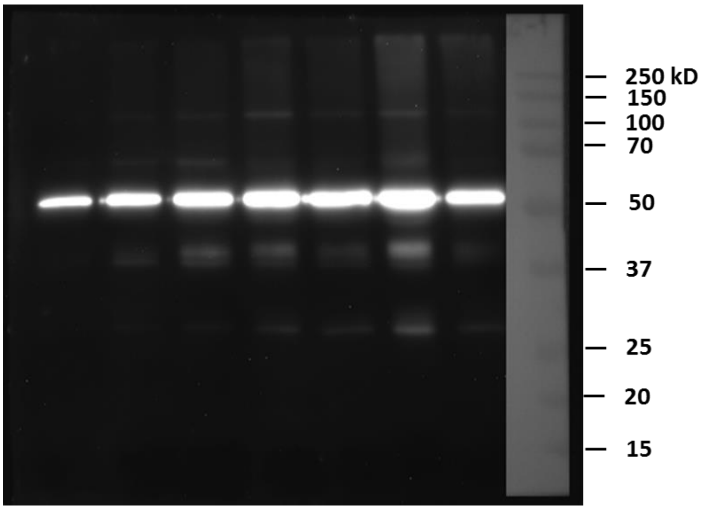

CBB

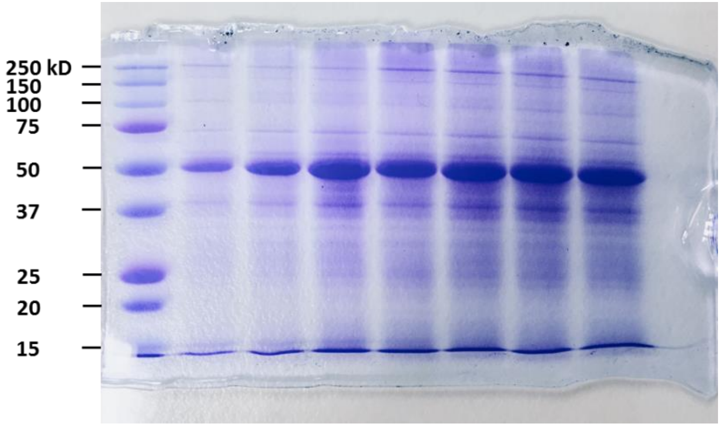

Supplement: Supplementary file 5 — Supplementary Data 5 [file 42003_2020_887_MOESM5_ESM.pdf]
