# Supplementary material for: Maize GOLDEN2-LIKE genes enhance biomass and grain yields in rice by improving photosynthesis and reducing photoinhibition
Source: Commun Biol. 2020 Apr 1;3:151. doi: 10.1038/s42003-020-0887-3 (PMC7113295; doi:10.1038/s42003-020-0887-3)
Supplement: Supplementary file 6 — Supplementary Information [file 42003_2020_887_MOESM6_ESM.pdf]

Supplemental information

Supplementary Tables

Supplementary Table 1: Maximum rate of carboxylation (V_{cmax}) and electron transport (J_{max}), rate of triose-phosphate utilization (TPU) and mitochondrial respiration (R_d) in WT and transgenic lines grown in the field in Beijing, 2019.

	V_{cmax}[a] (μmol m^{-2} s^{-1})	J_{max}[a] (μmol m^{-2} s^{-1})	TPU (μmol m^{-2} s^{-1})	R_d (μmol m^{-2} s^{-1})
WT	31.96±0.51	55.04±2.37	11.31±0.16	3.03±0.05
ZmUBI$_{pro}$:ZmGLK1-2	33.14±0.44	62.49±2.64	11.59±0.49	3.28±0.51
ZmUBI$_{pro}$:ZmGLK1-3	33.53±0.83	60.22±2.69	11.90±0.16	2.94±0.49
ZmUBI$_{pro}$:ZmG2-2	36.19±0.34 **	70.39±0.99 *	12.23±0.19 *	3.53±0.27
ZmUBI$_{pro}$:ZmG2-3	38.21±0.38 **	69.81±2.93 *	13.52±0.29 **	4.17±0.60

[a]: V_{cmax} and J_{max} were estimated at 25 °C.

Data are mean ± SE (n = 3 biological replicates). *$P < 0.05$, **$P < 0.01$ compared with WT according to two-tailed Student's *t*-test.

Supplementary Table 2: Seed yield in *ZmUBI*$_{pro}$*:ZmGLK1* and *ZmUBI*$_{pro}$*:ZmG2* transgenic lines in Beijing and Hainan.

	Seed yield per plant (g) [a]	Seed yield per plot (g) [b]
WT	21.52±2.58	689.3 ± 79.8
ZmUBI$_{pro}$*:ZmGLK1-2*	25.16±1.99	1080.3 ± 19.0 *
ZmUBI$_{pro}$*:ZmGLK1-3*	29.78±2.37 *	998.33 ± 86.7 *
ZmUBI$_{pro}$*:ZmG2-2*	33.34±2.66 *	1387.3 ± 36.4 **
ZmUBI$_{pro}$*:ZmG2-3*	35.31±2.51 **	1505.7 ± 110.8 **

(**a**) Seed yield per plant was obtained from field experiments in Beijing, May 2017 to September 2017. Data are mean ± SE (n = 5 biological replicates). (**b**) Seed yield per plot was calculated from 30 independent rice plants within a plot, and three plots that were placed randomly in the field experiment in Hainan, December 2018 to April 2019. Data are mean ± SE (n = 3 biological replicates). *$P < 0.05$, **$P < 0.01$ compared with WT according to two-tailed Student's *t*-test.

Supplementary Table 3: Primers used in this study.

Primer name	Sequence (5' to 3')	Usage
ZmG2-cloningF	GGGGACAAGTTTGTACAAAAAAGCAGGCTATGCTTGAGGTGTCGACGCTG	Vector construction
ZmG2-cloningR	GGGGACCACTTTGTACAAGAAAGCTGGGTTAGTATGTCATCCGGTGGCGC	
ZmGLK1-cloningF	GGGGACAAGTTTGTACAAAAAAGCAGGCTATGCTTGCAGTGTCGCCGTC	
ZmGLK1-cloningR	GGGGACCACTTTGTACAAGAAAGCTGGGTTCATCCACAAGCTTGGGCAC	
pVec8F (UBIpro)	TTTAGCCCTGCCTTCATACG	
pVec8R (nosT)	ATTGCCAAATGTTTGAACGA	
Hyg-F	ACGGTGTCGTCCATCACAGTTTGCC	Hygromycin resistance
Hyg-R	TTCCGGAAGTGCTTGACATTGGGGA	
HygProbe-F	CTTCTACACAGCCATCGGTC	Probe of DNA gel blot
HygProbe-R	CCGATGGTTTCTACAAAGATCG	
ZmG2-F	CATGGTGGACGACAACCTC	qRT-PCR
ZmG2-R	CACATGTTTGCTCCAACGAC	
ZmGLK1-F	GGACCTGGATTTCGACTTCA	
ZmGLK1-R	CACTCCCCTTTCCCTTCTTC	
OsActin-F	GGCACCACACCTTCTACAAT	
OsActin-R	CTCACACCATCACCAGAGT	
OsGLK1-F	AGCTGCGAGATTTCCTGCTC	
OsGLK1-R	ATAGCTGCGTCGATGCTCTC	
OsGLK2-F	AGGGGAGAGATTTTGGGATGC	
OsGLK2-R	TTCCTTCACGTCTTCCTTGG	

Supplementary Figures

Supplementary Figure 1

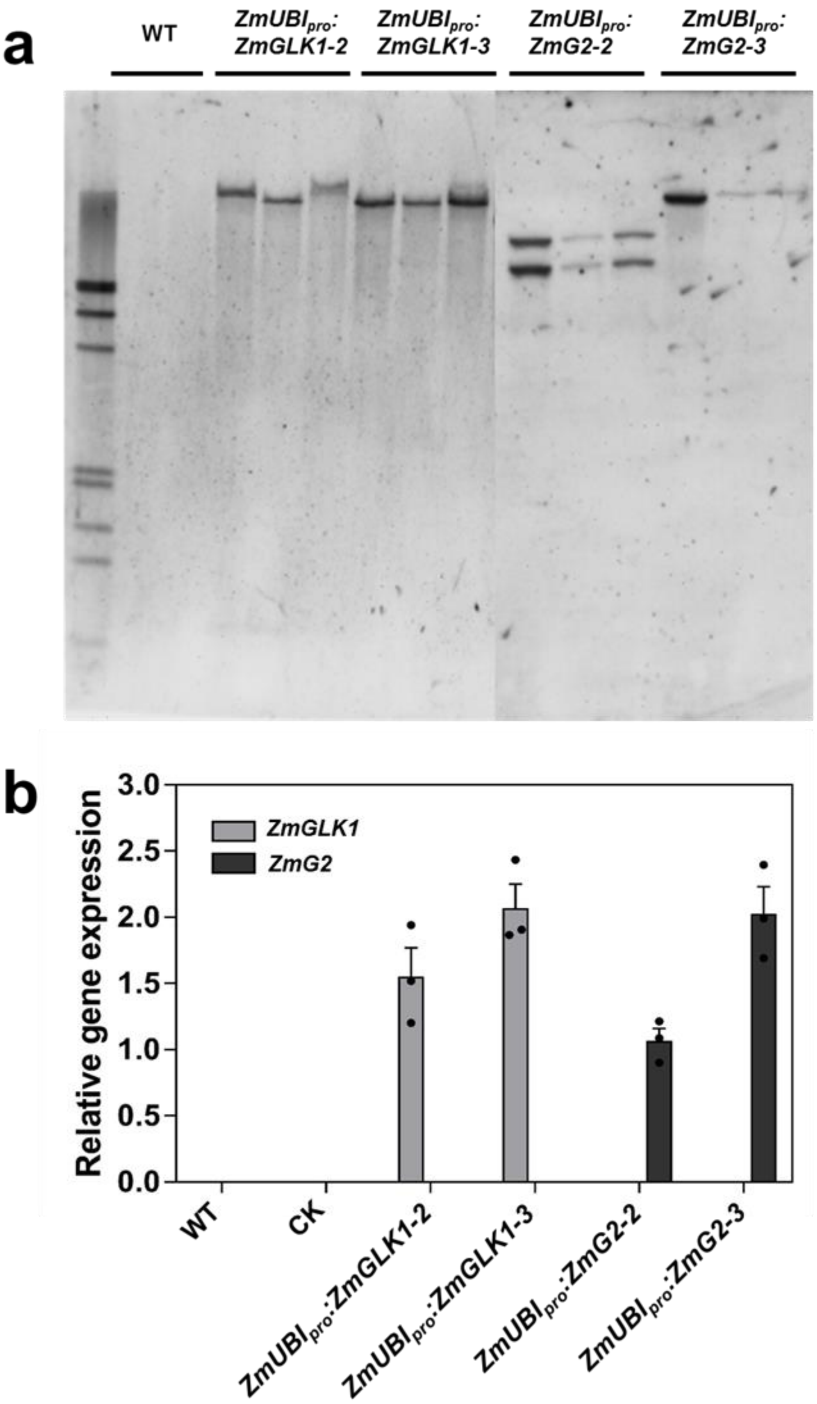

Supplementary Figure 1. Transgene copy number and relative transcript levels of *ZmGLK1* and *ZmG2* in transgenic lines.

(**a**) Transgene copy number in WT and two independent T5 transgenic lines transformed with *ZmUBI*$_{pro}$:*ZmGLK1* or *ZmUBI*$_{pro}$:*ZmG2* was detected by DNA gel

blot analysis of BglII digested genomic DNA. Each band represents a single transgene insertion event. (**b**) Relative transcript levels of *ZmGLK1* and *ZmG2* in WT and transgenic lines. In each case, RNA was isolated from 5th leaves of 3-week old seedlings grown hydroponically. Transcript levels were measured by qRT-PCR and quantified relative to actin transcript levels. Data are mean ± SE (n = 3 biological replicates), each dot represents a biological replicate. CK = null segregants isolated in the T4 generation from selfed heterozygous transgenic plants.

Supplementary Figure 2

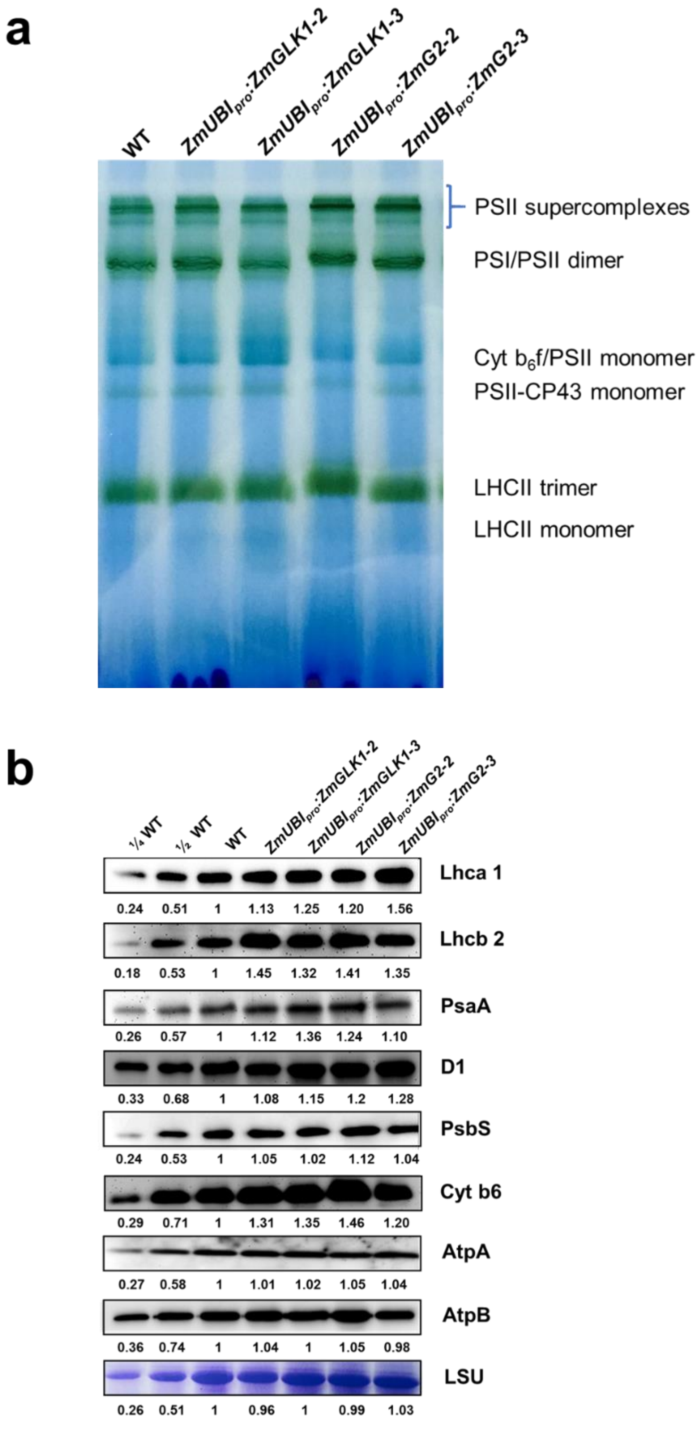

Supplementary Figure 2. Blue native (BN)-PAGE and immunoblot analyses of photosynthetic pigment-containing complexes.

(**a**) BN-PAGE analysis of photosynthetic pigment-containing complexes of WT and transgenic lines. Thylakoid membranes (10 μg chlorophyll per lane) from leaves of WT plants and *ZmUBI$_{pro}$:ZmGLK1* or *ZmUBI$_{pro}$:ZmG2* transgenic lines were solubilized with 2% dodecyl-β-D-maltoside and separated by 4-13% BN PAGE. The identities of

the resolved color bands are indicated.

(**b**) Immunoblot analysis of photosynthetic membrane proteins from WT and transgenic lines. Total proteins were extracted from leaves of young seedlings. 15 μg protein was loaded and separated by SDS-PAGE. A dilution series containing total protein was loaded in the lanes marked by 0.25, 0.5, WT, and transgenic lines, respectively. Western blot analysis was carried out with the antisera indicated. The Rubisco large subunit (LSU) was used as a loading control. The number below the gel lane represented the relative protein level, which was quantified from the band intensity using ImageJ software, and normalized relative to the WT.

Supplementary Figure 3

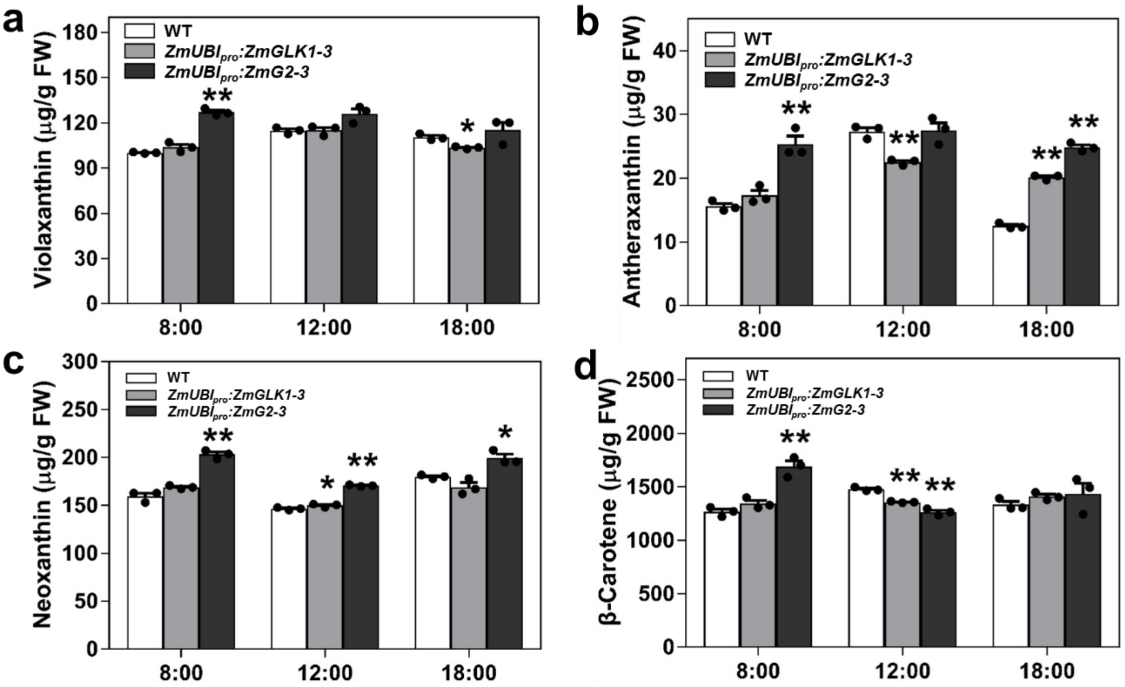

Supplementary Figure 3. Diurnal variation of carotenoid composition of leaf tissue from WT and transgenic lines in the field.

(**a**-**d**) Diurnal change of violaxanthin (**a**), antheraxanthin (**b**), neoxanthin (**c**) and β-carotene (**d**) content. All pigments were measured in flag leaves sampled at the heading stage at 8 am, 12 am and 6 pm from the field experiment in Beijing, 2019. Data are mean ± SE (n = 3 biological replicates), each dot represents a biological replicate. *$P < 0.05$, **$P < 0.01$ compared with WT according to two-tailed Student's *t*-test.

Supplementary Figure 4

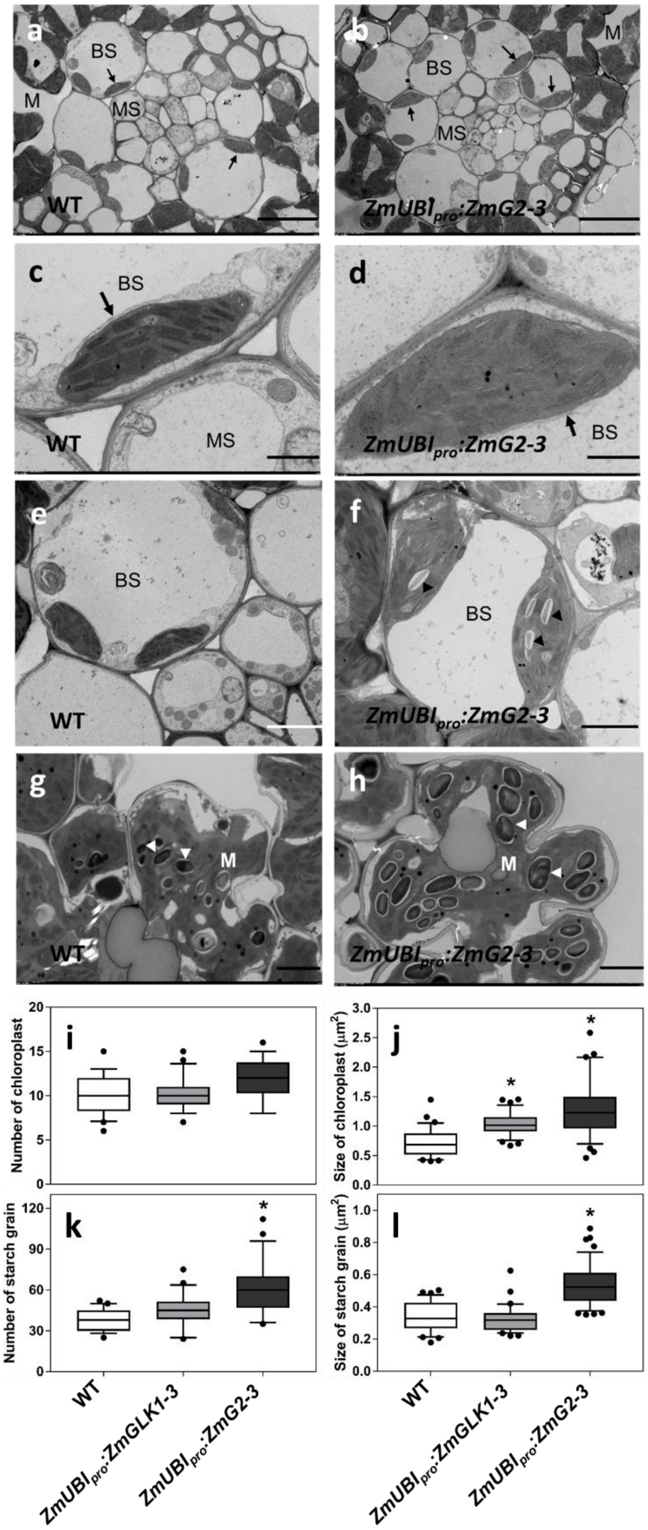

Supplementary Figure 4. Chloroplasts in *ZmUBI$_{pro}$:ZmG2* transgenic plants accumulate significantly more starch than wild type.

(**a-h**) Transmission electron micrographs of chloroplasts in leaves of WT (**a**, **c**, **e**, **g**) and *ZmUBI$_{pro}$:ZmG2-3* (**b**, **d**, **f**, **h**) plants. Low magnification showing arrangement of

bundle sheath (BS), mestome sheath (MS) and mesophyll (M) chloroplasts around veins (**a**, **b**); higher magnification of bundle sheath chloroplasts (**c**, **d**); starch grains in bundle sheath chloroplasts (**e**, **f**), in recent fully expanded leaves of 4-week-old rice seedlings grown hydroponically and harvested 4 h after dawn. Mesophyll chloroplasts in flag leaves during the heading stage in the Hainan field (**g**, **h**). Arrows in (**a-d**) point to chloroplasts, arrowheads in (**e-h**) point to starch grains. Scale bars = 10 μm (**a-b**); 1 μm (**c-d**); 2 μm (**e-h**). (**i-l**) Quantification of number (**i**) and size (**j**) of bundle sheath chloroplasts, and of number **(k)** and size **(l)** of starch grains in mesophyll cell chloroplasts. Starch grains were quantified in flag leaves harvested at 11 am (4 h after dawn) during the heading stage in the Hainan field, 2018. Number of chloroplasts were quantified per bundle sheath cell and starch grains were quantified per unit area mesophyll cell. Box and whisker plots show median (line) and outliers (•). (n > 20 cells), *$P < 0.05$, **$P < 0.01$ compared with WT according to Student's t-test.

Supplementary Figure 5

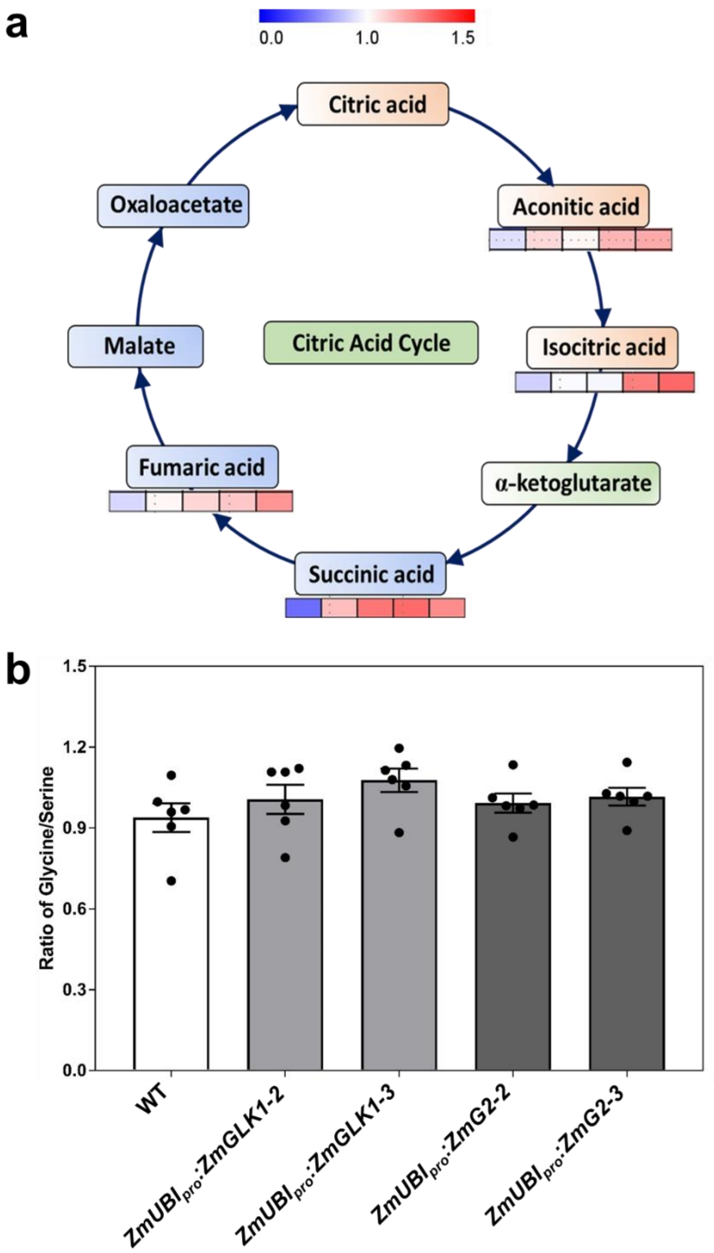

Supplementary Figure 5. Metabolite profiling of WT and transgenic lines.

(**a**) Changes in four metabolites of the citric acid cycle are visualized in a pathway map. Citric acid, α-ketoglutarate, malate and oxaloacetate were not detected. Metabolite content is presented as median-centered averages with 6 biological replicates each, in the same order as Fig. 4e. Red and blue colors indicate high and low content, respectively. (**b**) Ratio of glycine: serine calculated by the relative content of glycine and serine. Data are mean ± SE (n = 6 biological replicates), each dot represents a biological replicate.

Supplementary Figure 6

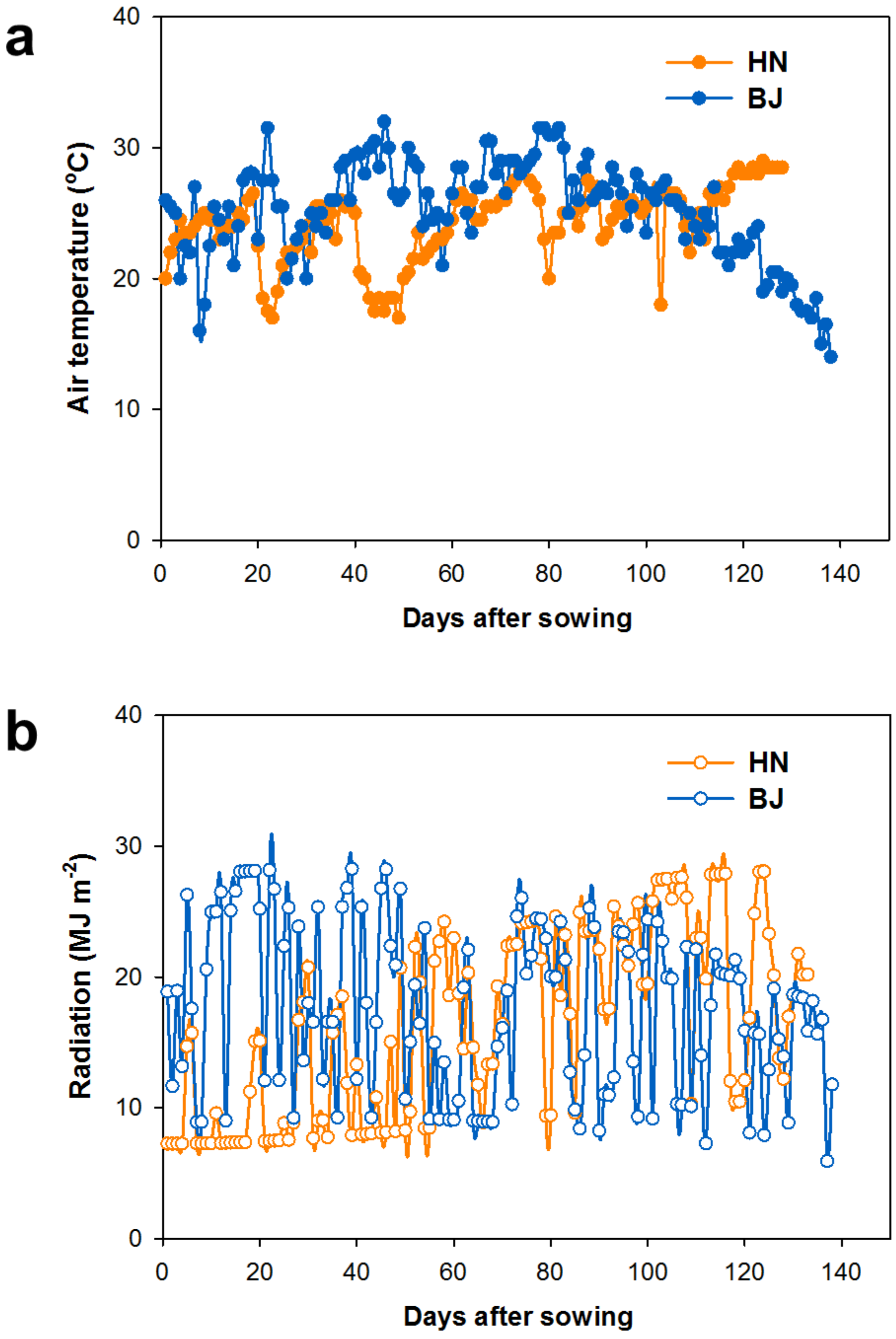

Supplementary Figure 6. Meteorological data.

(**a**, **b**) Air temperature (**a**) and solar radiation (**b**) during the growing seasons at the experimental sites in Hainan (HN, December 2017 – April 2018) and Beijing (BJ, May 2018 – September 2018).

Supplementary Figure 7

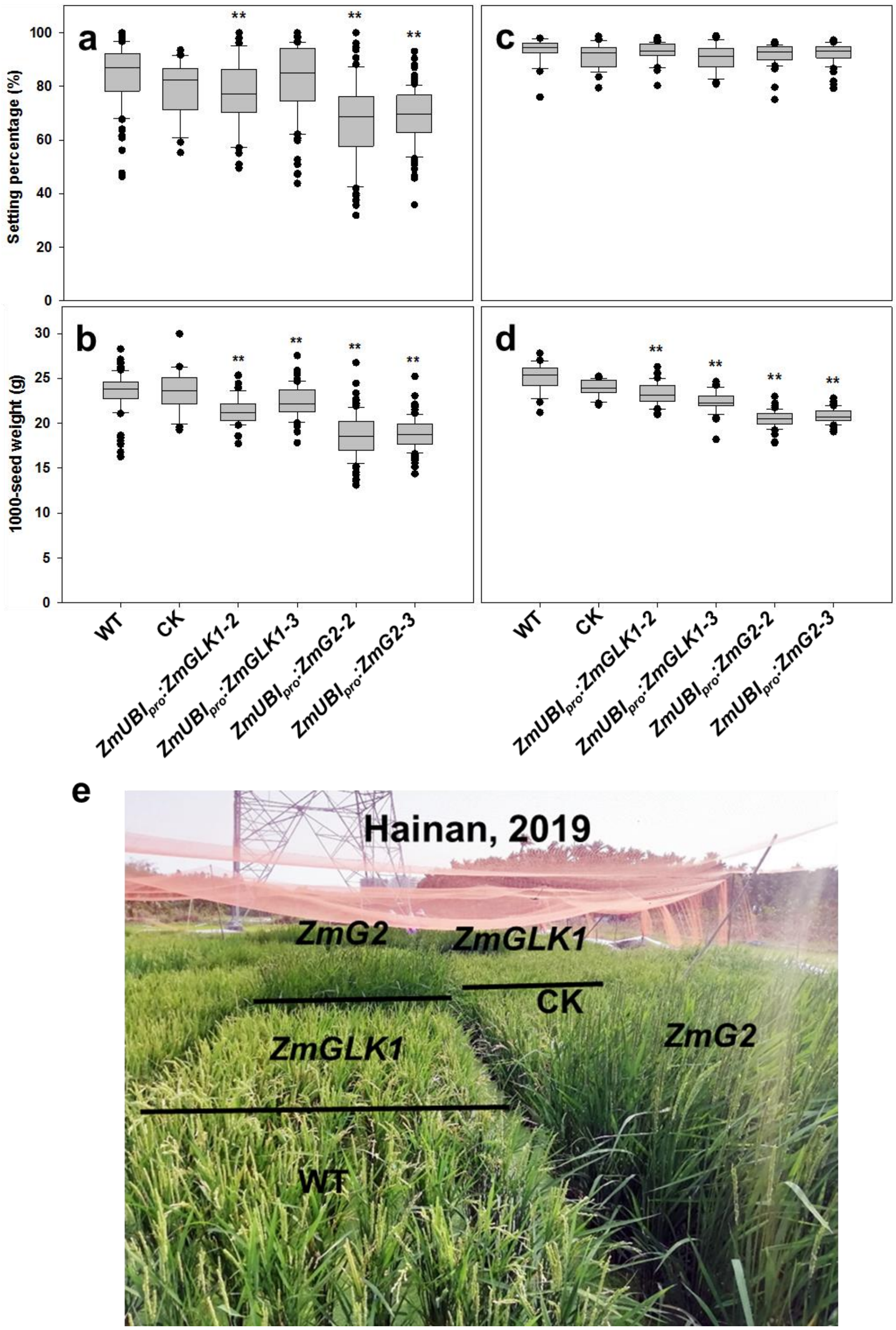

Supplementary Figure 7. Yield parameters and phenotype for WT and transgenic lines in the field.

(**a**, **b**) Yield parameters from field experiments in Beijing, May 2018 to September 2018.

(**c**, **d**) Yield parameters from field experiment in Hainan, December 2017 to April 2018. All data were calculated from at least 20 independent rice plants. CK = null segregants isolated from selfed heterozygous transgenic plants. Box and whisker plots show median (line) and outliers (•). *$P < 0.05$, **$P < 0.01$ compared with WT according to Student's *t*-test. (**e**) Growth of WT and transgenic plants under natural short-day field conditions in Hainan, 2019.

Supplementary Figure 8

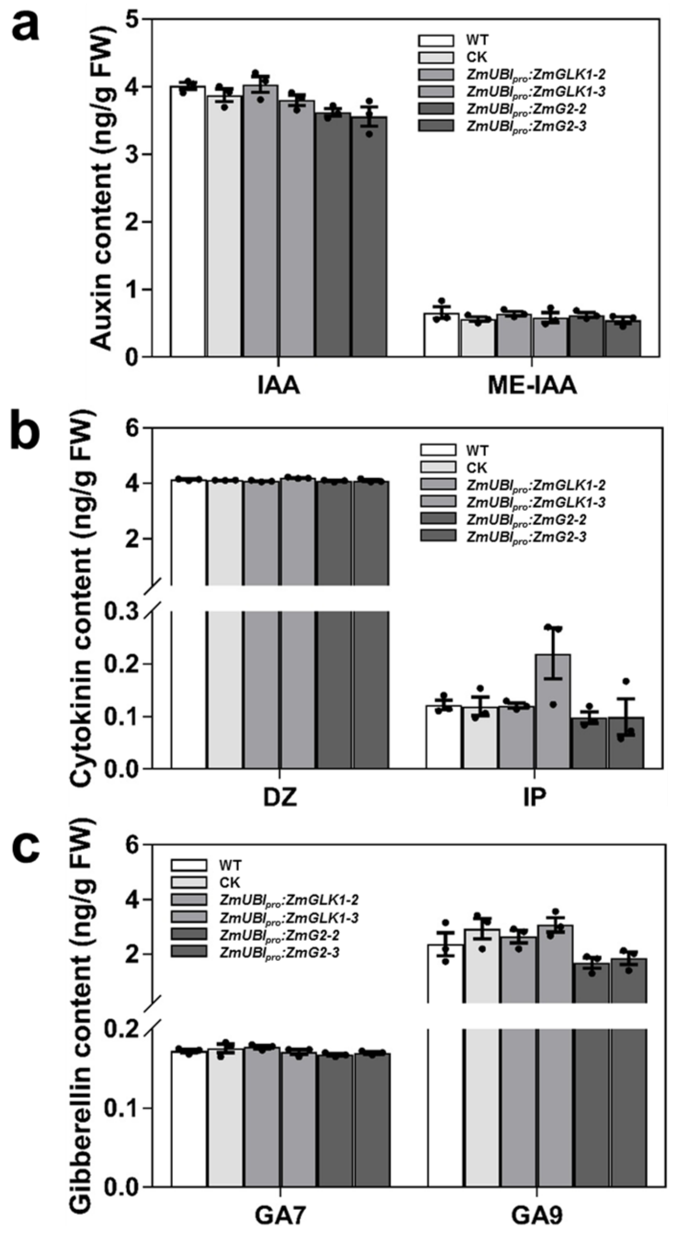

Supplementary Figure 8. Hormone content in flag leaves of WT and transgenic lines at heading stage in the field, Beijing.

(**a**) Auxin content. IAA = indole-3-aceticacid, ME-IAA = methylindole-3-acetate. (**b**) Cytokinin content. IP = N6-isopentenyladenine, DZ = dihydrozeatin. (**c**) Gibberellin content. GA = gibberellin. Data are mean ± SE (n = 3 biological replicates), each dot represents a biological replicate. CK = null segregants isolated from selfed heterozygous transgenic plants.
