# Supplementary material for: Maize GOLDEN2-LIKE genes enhance biomass and grain yields in rice by improving photosynthesis and reducing photoinhibition
Source: Commun Biol. 2020 Apr 1;3:151. doi: 10.1038/s42003-020-0887-3 (PMC7113295; doi:10.1038/s42003-020-0887-3)
Supplement: Supplementary file 7 — Description of Additional Supplementary Files [file 42003_2020_887_MOESM7_ESM.pdf]

Descriptions of Additional Supplementary Files

Supplementary data 1: Data for Figure 1a-f, Figure 2a-j, Figure 3a-h, Figure 4a-e

Supplementary data 2: Data for Figure 5a-f, Figure 6a-e

Supplementary data 3: Data for Figure 5g-l, Figure 6f-j

Supplementary data 4: Data for Supplementary Table 1, Supplementary Table 2, Supplementary Figure 1b, Supplementary Figure 3a-d, Supplementary Figure 4i-l, Supplementary Figure 5a-b, Supplementary Figure 6a-b, Supplementary Figure 7a-d, Supplementary Figure 8a-c

Supplementary data 5: Data for Figure 2k, Supplementary Figure 2b
